# Supplementary material for: Lasso peptides sviceucin and siamycin I exhibit anti-virulence activity and restore vancomycin effectiveness in vancomycin-resistant pathogens
Source: iScience. 2025 Jan 30;28(3):111922. doi: 10.1016/j.isci.2025.111922 (PMC11872507; doi:10.1016/j.isci.2025.111922)
Supplement: Document S1. Figures S1–S6 and Tables S1–S7 [file mmc1.pdf]

iScience, Volume *28*

Supplemental information

Lasso peptides sviceucin and siamycin I exhibit anti-virulence activity and restore vancomycin effectiveness in vancomycin-resistant pathogens

Abdelhakim Boudrioua, Benjamin Baëtz, Solenn Desmadril, Christophe Goulard, Anne-Claire Groo, Carine Lombard, Sabrina Gueulle, Marie Marugan, Aurélie Malzert-Fréon, Axel Hartke, Yanyan Li, and Caroline Giraud

Supplemental figures

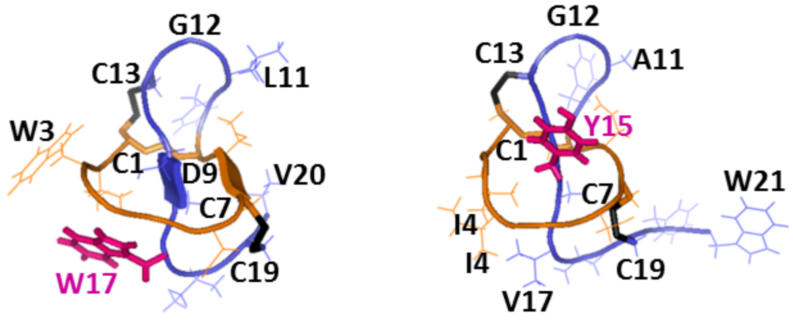

Figure S1. Three-dimensional structures of class I lasso peptides, Related to Figure 1.

Left: sviceucin[1] (PDB: 2LS1); Right: RP-71955[2] (PDB: 1RPC) which is a close homolog of siamycin I (primary sequence: CLGIGSCNDFAGCGYAVVCFW).

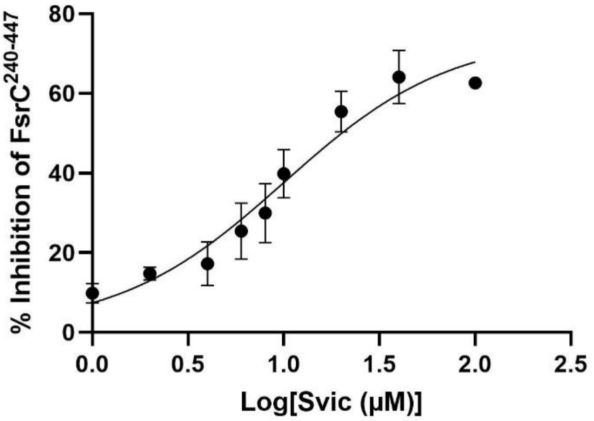

Figure S2. Inhibition of the autophosphorylation activity of FsrC in the presence of sviceucin, Related to Figure 1. For this, the cytoplasmic region of FsrC ($FsrC^{240-447}$) was produced recombinantly with a fusion to a Maltose binding protein (MBP) at the N-terminus. Data presented are means from three independent replicates. The dose-response curve is shown and the data was analyzed by nonlinear regression fit function in GraphPad, which gives an IC_{50} of 10.1 +-4.7 µM. Error bars represent the standard deviation.

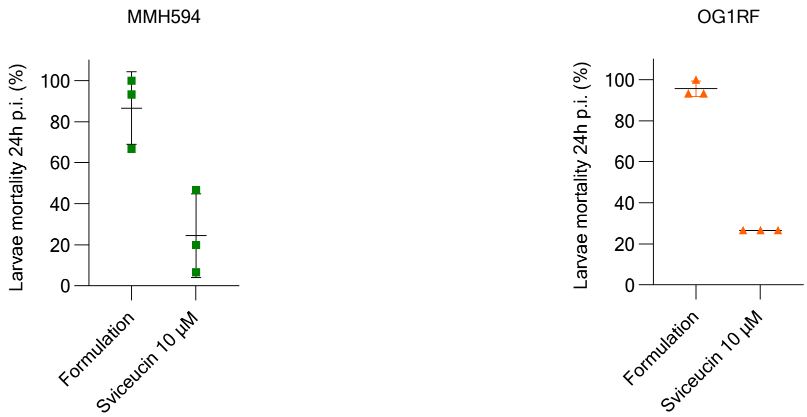

Figure S3. Survival of *Galleria mellonella* larvae infected with *E. faecalis* MMH594 or OG1RF treated with sviceucin, Related to Figure 1. Data presented are means from three independent experiments as indicated by individual data points and error bars represent the standard deviation.

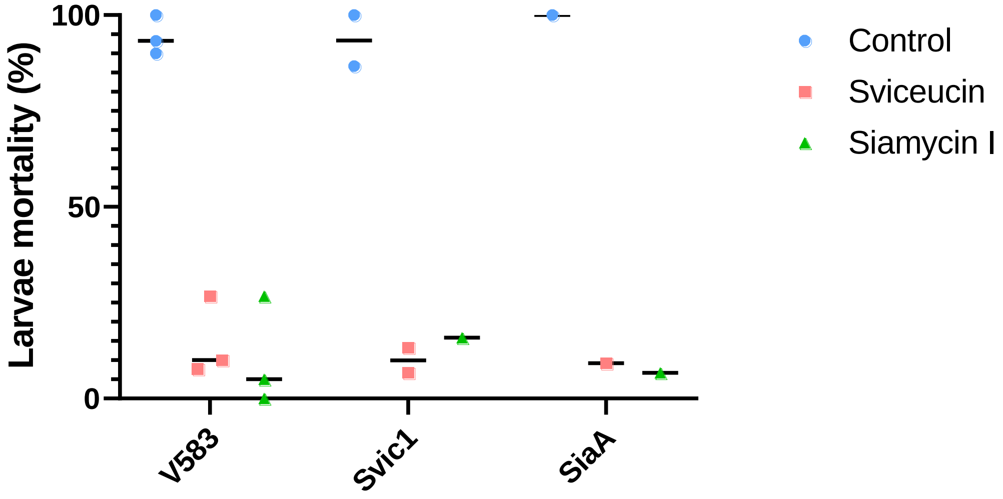

Figure S4. Anti-virulence activity of sviceucin and siamycin I on V583, Svic1 and SiaA, Related to Table 2. Mortality of *Galleria mellonella* larvae 24 h post-infection (p.i.) with *E. faecalis* V583, mutant Svic1 and mutant SiaA, pre-treated with either siamycin I (2 µM), sviceucin (10 µM) or the 1:1 water:methanol peptide formulation without peptide (control). Data presented are means from independent experiments as indicated by individual data points.

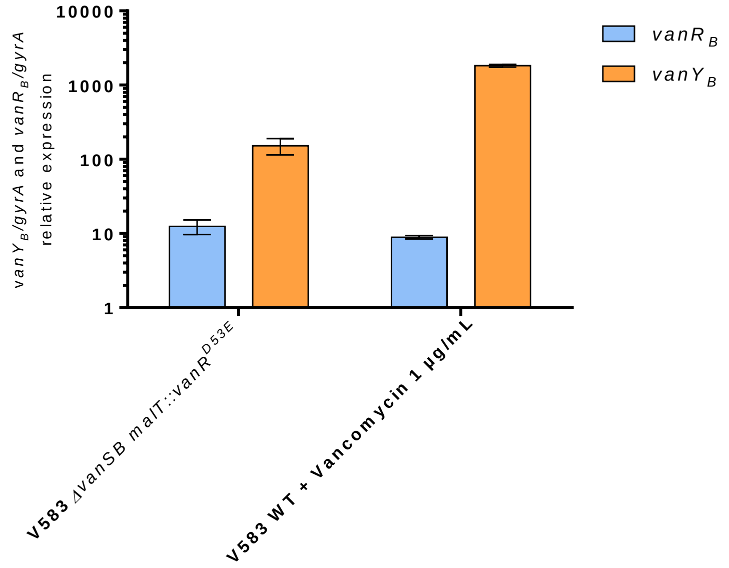

Figure S5. Relative expression of *vanY*$_B$ in *E. faecalis* V583 Δ*vanSB malT::vanRD53E* and in V583 in presence of vancomycin (1 µg/mL) , Related to Table 3. Data presented are means from three independent experiments and error bars represent the standard deviation.

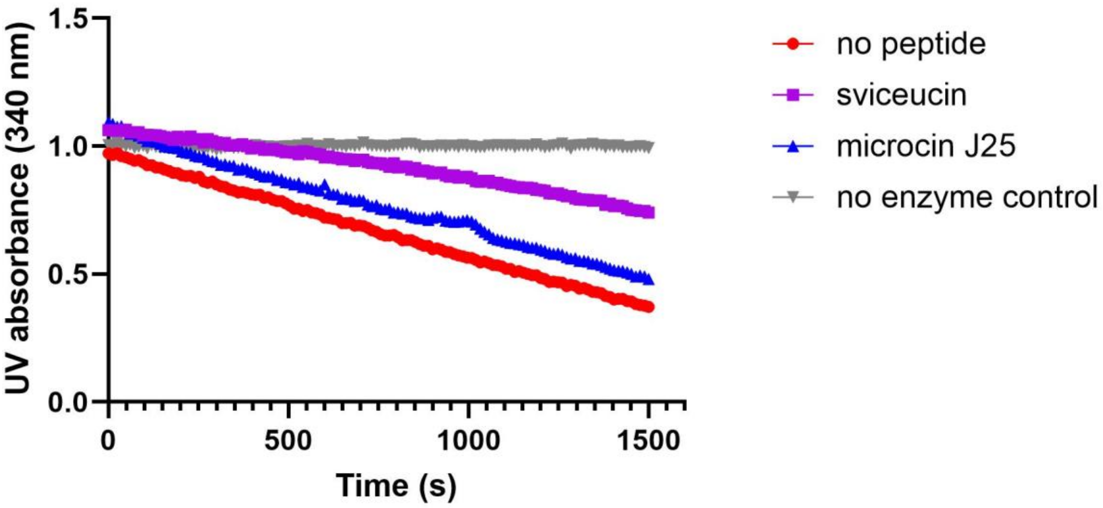

Figure S6. Autophosphorylation activity of VanS$^{122\text{-}384}$ in the presence of lasso peptides, Related to Figure 3.

A continuous assay with coupling enzymes was performed as previously described[3]. Reaction rate was reflected by the consumption of NADH monitored by UV spectroscopy at 340 nm.

Supplemental tables

Table S1. Sviceucin and siamycin I reverse vancomycin resistance in a dose dependent manner, Related to Table 1. MICs of vancomycin alone, in combination with sviceucin, or with siamycin I, for *E. faecalis* V583, determined by checkerboard broth microdilution assays.

	Vancomycin MIC (µg/mL)
control	32
sviceucin 2.5 µM	8
sviceucin 5 µM	4
sviceucin 10 µM	2
siamycin I 1µM	4
siamycin I 2 µM	2
siamycin I 5 µM (inhibitory concentration)	< 0.5

Table S2. Characteristics of the spontaneous mutants resistant to lasso peptides and vancomycin combinations in comparison to the parental strain, Related to Table 2.

Clone no.	Isolation method	Position in V583 genome	V583 reference	Mutation	Mutations consequences	Vancomycin MIC (µg/ml)	Vancomycin + sviceucin MIC (µg/ml)	Vancomycin + siamycin I MIC (µg/ml)
V583	n.a.	n.a.	n.a.	n.a.	parental strain	32	2	2
Svic1	5*10^8 plating on vancomycin (32 µg/ml) and sviceucin (10 µM)	2216922	C	A	VanSB S402I	128	128	2
Svic2		2527311	C	A	nonsense mutation in *atpA*	256	256	2
Svic3		2529182	C	A	nonsense mutation in *atpF*	256	256	2
Svic4		2525799	C	T	*atpD* RBS modification	256	256	2
SiaA	serial passages	2216933	A	C	VanSB D398E	128	128	32
SiaB		1690453	T	A	CcpA D121V	64	32	32
SiaC		1690372	G	A	CcpA A148V	64	32	32
SiaD		1690550	C	G	CcpA A89P	64	32	32
SiaE		1690580	A	deletion	frameshift in *ccpA*	32	16	16

Table S3. Strains used for this study, Related to STAR Methods. DHR = Double Homologous Recombination, C = Conjugation, R = Resistant, S = Sensitive, V = Variable

Strain	Description	Origin
E. faecalis		
V583	Clinical isolate, PAI (esp^{-}, cyl^{-}), van^{RB}	Sahm *et al.*, 1989[4]
V583 Δ*vanB*	DHR, van^{S}	This study
V583 Svic1	Spontaneous mutant, van^{RB}, svi^{R}, $vanS_B^{S402I}$	This study
V583 Svic1 Δ*vanB*	DHR, van^{S}, $vanS_B^{S402I}$	This study
V583 SiaA	Spontaneous mutant, van^{RB}, svi^{R}, sia^{R}, $vanS_B^{D398E}$	This study
V583 $vanS_B^{S402I}$	DHR, van^{RB}, svi^{R}, $vanS_B^{S402I}$	This study
V583 $vanS_B^{D398E}$	DHR, van^{RB}, svi^{R}, sia^{R}, $vanS_B^{D398E}$	This study
V583 Δ$vanS_B$::*vanS*	DHR, van^{S}, Δ$vanS_B$::*vanS*	This study
V583 Δ$vanR_BS_B$::*vanRS*-P_{vanY}	DHR, van^{R}, svi^{R}, Δ$vanR_BS_B$::*vanRS*-P_{vanY}	This study
V583 Δ*vanB*::*vanA*	DHR, van^{RB}, Δ*vanB*::*vanA*	This study
V583 Δ$vanS_B$	DHR, van^{V}, Δ$vanS_B$	This study
V583 Δ$vanS_B$ *malT*::$vanR^{D53E}$	DHR, van^{RB}, (svi^{R}, sia^{R}) Δ$vanS_B$, *malT*::$vanR^{D53E}$	This study
MMH594	Clinical isolate, PAI+, van^{S}	Shankar *et al.*, 2002[5]
OG1RF	Human isolate, PAI-, van^{S}	Dunny *et al.*, 1978[6]
JH2-2 08048	C, van^{RB}, *E. faecium* 08/048 van^{RB} plasmid	Ladjouzi *et al.*, 2013[7]
Merz96	Clinical isolate, van^{RB}	Harrington *et al.*, 2004[8]
1 231 410	Clinical isolate, van^{R}	Broad Institute
HIP11704	Clinical isolate, Van^{R}	Broad Institute
E. faecium		
1 231 502	Clinical isolate, van^{R}	Broad Institute
1 230 933	Clinical isolate, van^{R}	Broad Institute
S. aureus		
T-SAR12 VRSA.B	C, van^{RB}, *E. faecalis* 08048 van^{RB} plasmid	This study
T-SAR12 VRSA.A	C, van^{RA}, *E. faecalis* HIP11704 van^{RA} plasmid	This study
16038	Clinical isolate, VISA	Caen University Hospital
E. coli		
Top10F'	F´{lacIq Tn10 (TetR)} mcrA Δ(mrr-hsdRMS-mcrBC) Φ80lacZΔM15 ΔlacX74 recA1 araD139 Δ(ara-leu)7697 galU galK rpsL endA1 nupG	ThermoFisher Scientific
EC1000	Δ(araA-leu)7697, *araD139+B/r, Δ(codB-lacI)3, galK16, galE15(GalS), λ-, e14-, relA1, rpsL150(strR), spoT1, mcrB1	Leenhouts *et al.*, 1996[9]
NEB5α™	fhuA2 Δ(argF-lacZ)U169 phoA glnV44 Φ80 Δ(lacZ)M15 gyrA96 recA1 relA1 endA1 thi-1 hsdR17	New England BioLabs®
BL21(DE3)	F⁻ ompT hsdSB (rB-mB-) gal dcm (DE3)	ThermoFisher Scientific

Table S4. Plasmids used for this study, Related to STAR Methods.

Plasmid	Description	Origin
pEBM2	oriT, *repA* TS, lac promoter, lac operator, *lacZ*, *thyA**, Cm^R<br>For *E. coli* : replicative<br>For *E. faecalis* : replicative at 30°C and integrative at 37°C	Lab collection
pEBM2 Δ$vanS_B$	pEBM2-($vanR_B$-$VanY_B$)	This study
pEBM2 Δ$vanS_B$::*vanS*	pEBM2-($vanR_B$-*VanS*-$VanY_B$)	This study
pEBM2 Δ$vanR_BS_B$::*vanRS*-P_{vanH}	pEBM2-($vanR_B$ upstream-*vanR*-*vanS*-P_{vanH}-$vanY_B$)	This study
pEBM2 *malT*::P_{hup}-$vanR^{D53E}$	pEBM2-(start *malt*-P_{hup}-$vanR^{D53E}$-end *malt)*	This study
pAS222	Tet^R, Amp^R, repA-pG+host4<br>For *E. coli* : replicative<br>For *E. faecalis* : replicative at 30°C and integrative at 37°C	Jönsson *et al.*, 2009[10]
PAS222 $vanS_B^{S402I}$	pAS222-($vanR_B$-$vanS_B^{S402I}$-$vanY_B$*)*	This study
PAS222 $vanS_B^{D398E}$	pAS222-($vanR_B$-$vanS_B^{D398E}$-$vanY_B$*)*	This study
pWS3	pG^+host9 derivative, $spectinomycin^R$, $erythromycin^S$<br>Suicide vector in *E. faecalis*	Zhang *et al.*, 2011[11]
pWS3 Δ*vanB*	pWS3-(*vanHB-vanXB*)	This study
pWS3 Δ*vanB::vanA*	pWS3-(*vanHB-vanA-vanXB*)	This study
pET29b(+)	Kan^R, T7lac, His-tag, S-tag, cleavage of tags by thrombin	Novagen
pET29b(+) $vanS^{127-384}$	Heterologous production in *E. coli*	This study
pHisMBPTev	Modification based on pMAL-c5e vector (NEB Biolabs)	home-made vector collection
pHisMBPTev $fsrC^{240-447}$	Heterologous production in *E. coli*	This study

Table S5. Primers used for this study, Related to STAR Methods.

Primers	Sequence	Description	Used in
OCG248	ATCAAGCTTATCGATACCGT	pEBM2 amplification ; pEBM2-upstream $vanR_B$	pEBM2 $\Delta vanS_B$; pEBM2 $\Delta vanS_B$::*vanS* ; pEBM2 $\Delta vanR_BS_B$::*vanRS* -P_{vanH} ; pEBM2 *malT*::P_{hup}-$vanR^{D53E}$
OCG249	ATCGAATTCCTGCAGC	pEBM2 amplification	
OCG250	GGCGGATCCCCCGGGCTGCAGGAATTCGATTCGATACGAATTCTACTTGT	pEBM2-$vanR_B$	pEBM2 $\Delta vanS_B$
OCG244	GGTCAAAATCATTTAAGTTTTAAATATTTACATTATAATGATTCCTCCAA	$vanR_B$-$vanY_B$	
OCG104	CATTATAATGTAAATATTTAAAACTTAAATGATTTTGACC	$vanR_B$-$vanY_B$	
OCG251	CTCGAGGTCGACGGTATCGATAAGCTTGATTTGTATTCGGCGACTTT	$vanY_B$-pEBM2	
M13F	GTAAAACGACGGCCAG	Control	pEBM2 $\Delta vanS_B$; pEBM2 $\Delta vanS_B$ ::*vanS*
M13R	CAGGAAACAGCTATGAC	Control	
OCG242	AGGTTACCGATTGGAGGAATCATTATAATGTATATCGTTGCAATTGTTGT	$vanR_B$-*vanS*	pEBM2 $\Delta vanS_B$ ::*vanS*
OCG243	GGTCAAAATCATTTAAGTTTTAAATATTTAGGACCTCCTTTTATCAACCA	*vanS*-$vanY_B$	
OHB37	TTGGTTGATAAAAGGAGGTCCTAAATATTTAAAACTTAAATGATTTTGAC	pEBM2 $\Delta vanS_B$ amplification	
OCG106	GCAACGATATACATTATAATGATTCCTCCAATCGGTAACC		
OCG110	CGCGGTCGACGCACCAGATAATGATAATCCGAACCTGG	Control	
OCG111	CGCGGCGGCCGCCGGGAAGCGCTGTGTCAATCTTTTTCTG	Control	
OHB29	GCGCTATCCAATGTCATATTG	Control	
OHB30	AATCCAGCCAGAACAAAACG	Control	
OBB003	TATGACTTTGACGGTGATGGCA	Control	
OBB004	GCTGGTACGATTGTTATTATGGCAA	Control	
OHB27	AAACGCCGCTGCATACAGTGAG	Control	pEBM2 $\Delta vanS_B$ ::*vanS* ; pEBM2 $\Delta vanR_BS_B$ ::*vanRS* -P_{vanH}
OHB28	CGCTGGAAGCTCTACCCTAAAC	Control	
OBB056	GGACACAGACGAGCGGAAC	Control	
OBB057	AACTCGGTGGGAGTAAGGGA	Control	
OBB058	AGGGTAGAGCTTCCAGCGAT	Control	
OBB059	GTTTAAGGTCTTTGCCGCCAG	Control	
OSD5	CAGTCACGACGTTGTAAAACGACGGC	Control	
OSD6	AACAATTTCACACAGGAAACAGCTATGACCATGA	Control	
OBB041	GCTTTGAAACTACAGGGAAACTACAGACTGAGGGCTTCATTATACAGGAA	Upstream $vanR_B$-*vanR*	pEBM2 $\Delta vanR_BS_B$ ::*vanRS* -P_{vanH}
OBB016	CACATTGGAATGATAGTTGCTTTTTTCCATAATTAAGACCAACCCTTTCT	*vanS*-$vanY_B$	

OBB036	GGCGGATCCCCCGGGCTGCAGGAATTCGATGACTGAGGATGGGAATGTAT	pEBM2 upstream $vanR_B$ amplification	
OBB037	CTCGAGGTCGACGGTATCGATAAGCTTGATCAGTCTGTAGTTTCCCTGTA		
OBB039	GCTTTGAAACTACAGGGAAACTACAGACTGATGGAAAAAAGCAACTATCA	Upstream $vanR_B$-$vanY_B$	
OBB018	CTCGAGGTCGACGGTATCGATAAGCTTGATTGGTTGTATAACGCACAATC	$vanY_B$-pEBM2	
OBB038	CAGTCTGTAGTTTCCCTGTAGT	pEBM2 upstream $vanR_B$-$vanY_B$ amplification ; upstream $vanR_B$-$vanY_B$	
OBB040	ATGGAAAAAAGCAACTATCATTCC	pEBM2 upstream $vanR_B$-$vanY_B$ *amplification*	
OBB042	GTATCTCTTATAGAGGAGGTGAAATGATCCATGTCGATACGAATTCTACT	pEBM2-ΔP_{tuf}-$vanR^{D53E}$ amplification	pEBM2 $malT::vanR^{D53E}$
OBB043	GAATATCTAAAAAAAGTATGGTAGAATGCATGTTCTTGTTAACAATTCAA		
OBB044	TGCATTCTACCATACTTTTTTTAGAT	pEBM2-P_{hup}	
OBB045	GGATCATTTCACCTCCTCTA	P_{hup}-$vanR^{D53E}$	
OCG224	GTAATGCCCGCAGCGGG	Control	
OCG231	CCATTTCTTCCAGGTGTTGCG	Control	
OBB055	GAGTGGTCCAAAAACAGACAGCAAA	Control Control	
OCG199	TATTCTTGAGATTATGCTGCCCGGTATGAATG		
OCG225	GCGTTTGGGTCGCTATGGGAC	Control	
OSD5	CAGTCACGACGTTGTAAAACGACGGC	Control	
OSD6	AACAATTTCACACAGGAAACAGCTATGACCATGA	Control	
OHB31	TTCAATTCGCTCATAATTAATCC	pAS222 amplification	pAS222 $vanS_B^{D398E}$; pAS222 $vanS_B^{S402I}$
OHB32	AAAGGGGATTTTATGCGTGG		
OHB35	CCCTCGAGGTCCACGCATAAAATCCCCTTTATATCAAAAATCTGCGGGCG	pAS222-upstream $vanS_B^{398}$	
OHB36	GTTGTTAAAGACTCTCGCGAGTTTATCAGGATCGGACAGGACAATTTGTC	Upstream $vanS_B^{398}$-*vanS* C1	
OHB37	TTGGTTGATAAAAGGAGGTCCTAAATATTTAAAACTTAAATGATTTTGAC	*vanS* C1-downstream $vanS_B^{402}$	
OHB38	GAAAAAGGATTAATTATGAGCGAATTGAATGTCTCATTGGAAGCAGGAAC	Downstream $vanS_B^{402}$-pAS222	
OCG218	CGGTGATGGCAGCACAGTCC	Control	
OHB30	AATCCAGCCAGAACAAAACG	Control	
OHB29	GCGCTATCCAATGTCATATTG	Control	
OCG219	GCCGTCAAATCTCATTCCCGC	Control	
OHB26	TGAACGATGACCTCTAAT	Control	
M13F	GTAAAACGACGGCCAG	Control	

OCG104	CATTATAATGTAAATATTTAAAACTTAAATGATTTTGACC	Control	
OCG208	AGAAAAAGGATTAATTATGAGCGAATTGAACGGGAAGCGCTGTGTCAATC	Control	
OCG105	CTCTGCGCTTGTCTCATTGGAAGCAGG	Control	
OCG232	CACCACCACCACCACCAC	pET29b(+) amplification	pET29b(+) *vanS*$^{127\text{-}384}$
OCG233	CATATGTATATCTCCTTCTTAAAGTTAAACAAA		
OCG236	GTTTAACTTTAAGAAGGAGATATACATATGATGGATGTTATGGAACAAAA	pET29b(+)-*vanS*	
OCG237	AGCCGGATCTCAGTGGTGGTGGTGGTGGTGGGACCTCCTTTTATCAACCA	*vanS*-pET29b(+)	
OCG240	CGCGAAATTAATACGACTCACTATA	Control	
OCG241	GGGGTTATGCTAGTTATTGCTCA	Control	
OCG66	GCCAAGGTTGAACGGATGCAAGTTGCC	Upstream *vanB*	pWS3 Δ*vanB::vanA*
OCG67	TTCTATTCATGCTGTAAACTCCTTTCAAAGTTAAGACAAT	Upstream *vanB-vanA*	
OCG68	AGTTTACAGCATGAATAGAATAAAAGTTGCAATACTGTTT	Upstream *vanB-vanA*	
OCG69	TCCATACGGGTCACCCCTTTAACGCTAATACGATC	*vanA*-downstream *vanB*	
OCG70	AAAGGGGTGACCCGTATGGAAAATGGTTTTTTGTTTTTAG	*vanA*-downstream *vanB*	
OCG71	AGTCATGCAACCGTGACAATCAGCAGATTC	Downstream *vanB*	
OCG72	CGCGGTCGACCCTGACAGAGCAGCTACTTGACCTTC	Downstream *vanB*	pWS3 Δ*vanB ;* pWS3 Δ*vanB::vanA*
OCG73	CGCGGAATTCGGTACCGGGGAAATAATAGATTGCAAGC	Upstream *vanB*	
OCG92	TCCATACGGGGCTGTAAACTCCTTTCAAAGTTAAGACAAT	Upstream *vanB*	pWS3 Δ*vanB*
OCG93	AGTTTACAGCCCCGTATGGAAAATGGTTTTTTGTTTTTAG	Downstream *vanB*	
OCG268	CCGGGTCGACTCGAGCGGCC	pHisMBPTev amplification	pHisMBPTev *fsrC*$^{240\text{-}447}$
OCG269	ACCTTGGAAGTACAGGTTCTCCCCGA		
OCG272	AACCTCGGGGAGAACCTGTACTTCCAAGGTCTAGCGATGTTTCGTCATGA	pHisMBPTev-*fsrC*	
OCG273	GTCACGATGCGGCCGCTCGAGTCGACCCGGTCATTCGTTAACAACTTTTT	*fsrC*-pHisMBPTev	
OCG274	TTCTGGTATGCCGTGCGTACT	Control	
OCG275	TTGTCCTACTCAGGAGAGCGTT	Control	
2297L	TAGCGGAGAATCCAGAGGAA	*vanY*$_B$	*vanY*$_B$, RT-qPCR
2297R	GGGGTTCTGCCTGTTCACTA		
1818L	TAGGACAATCAGGTGCGTTG	*gelE*	*gelE*, RT-qPCR
1818R	CACTCTGAGTATCCGCACCA		
GyrAL	GATGGGGAAATCAGGGATTC	*gyrA*	*gyrA*, RT-qPCR
GyrAR	TCTTTTCCATTCGGCATTTC		

Table S6. Physicochemical characteristics of the uncharged and C1LP containing nanoemulsions, Related to STAR Methods.

	**Z-average diameter (nm)**	**PDI**	**Zeta potential (mV)**	**Vancomycin MIC on *E. faecalis* V583**
Blank NEs	54 ± 2	0.21 ± 0.02	-16 ± 4	32
Sviceucin I-NEs	52 ± 1	0.17 ± 0.03	-15 ± 5	4
Siamycin I-NEs	51 ± 1	0.17 ± 0.01	-17 ± 1	4

Table S7. Buffers screened for DSF, Related to STAR Methods.

Buffers	Final concentration
Tris pH 7.5	Tris 50 mM
	Glycerol 15 %
Tris NaCl pH 7.5	Tris 50 mM
	Glycerol 15 %
	NaCl 150 mM
Tris $MgCl_2$ pH 7.5	Tris 50 mM
	Glycerol 15 %
	$MgCl_2$ 5 mM
Tris NaCl/$MgCl_2$ pH 7.5	Tris 50 mM
	Glycerol 15 %
	NaCl 150 mM
	$MgCl_2$ 5 mM
Potassium phosphate pH 7.5	Potassium phosphate 20 mM
	Glycerol 10 %
Potassium phosphate NaCl pH 7.5	Potassium phosphate 20 mM
	Glycerol 10 %
	NaCl 500 mM
Potassium phosphate $MgCl_2$ pH 7.5	Potassium phosphate 20 mM
	Glycerol 10 %
	$MgCl_2$ 5 mM
Potassium phosphate NaCl/$MgCl_2$ pH 7.5	Potassium phosphate 20 mM
	Glycerol 10 %
	NaCl 500 mM
	$MgCl_2$ 5 mM
Sodium phosphate pH 7.5	Sodium phosphate 20 mM
	Glycerol 10 %
Sodium phosphate NaCl pH 7.5	Sodium phosphate 20 mM
	Glycerol 10 %
	NaCl 100 mM
Sodium Citrate pH 7.5	Sodium citrate 50 mM
	Glycerol 10 %

HEPES pH 7.5	HEPES 50 mM<br>Glycerol 10 %
HEPES NaCl pH 7.5	HEPES 50 mM<br>Glycerol 10 %<br>NaCl 100 mM
HEPES $MgCl_2$ pH 7.5	HEPES 50 mM<br>Glycerol 10 %<br>$MgCl_2$ 5 mM
HEPES NaCl/$MgCl_2$ pH 7.5	HEPES 50 mM<br>Glycerol 10 %<br>NaCl 100 mM<br>$MgCl_2$ 5 mM
PIPES pH 7.5	PIPES 50 mM<br>Glycerol 10 %
MOPS pH 7.5	MOPS 50 mM<br>Glycerol 10 %
MOPS NaCl pH 7.5	MOPS 50 mM<br>Glycerol 10 %<br>NaCl 10 mM
MOPS $MgCl_2$ pH 7.5	MOPS 50 mM<br>Glycerol 10%<br>$MgCl_2$ 5 mM
MOPS NaCl/$MgCl_2$ pH 7.5	MOPS 50 mM<br>Glycerol 10 %<br>NaCl 10 mM<br>$MgCl_2$ 5 mM

References

1. Li, Y., Ducasse, R., Zirah, S., Blond, A., Goulard, C., Lescop, E., Giraud, C., Hartke, A., Guittet, E., Pernodet, J.-L., et al. (2015). Characterization of Sviceucin from *Streptomyces* Provides Insight into Enzyme Exchangeability and Disulfide Bond Formation in Lasso Peptides. ACS Chem Biol *10*, 2641–2649. https://doi.org/10.1021/acschembio.5b00584.

2. Fréchet, D., Guitton, J.D., Herman, F., Faucher, D., Helynck, G., Monegier du Sorbier, B., Ridoux, J.P., James-Surcouf, E., and Vuilhorgne, M. (1994). Solution structure of RP 71955, a new 21 amino acid tricyclic peptide active against HIV-1 virus. Biochemistry *33*, 42–50. https://doi.org/10.1021/bi00167a006.

3. Lee, D., Lee, Y., Hye Shin, S., Min Choi, S., Hyeon Lee, S., Jeong, S., Jang, S., and Kee, J.-M. (2023). A simple protein histidine kinase activity assay for high-throughput inhibitor screening. Bioorganic Chemistry *130*, 106232. https://doi.org/10.1016/j.bioorg.2022.106232.

4. Sahm, D.F., Kissinger, J., Gilmore, M.S., Murray, P.R., Mulder, R., Solliday, J., and Clarke, B. (1989). In vitro susceptibility studies of vancomycin-resistant *Enterococcus faecalis*. Antimicrob Agents Chemother *33*, 1588–1591. https://doi.org/10.1128/aac.33.9.1588.

5. Shankar, N., Baghdayan, A.S., and Gilmore, M.S. (2002). Modulation of virulence within a pathogenicity island in vancomycin-resistant *Enterococcus faecalis*. Nature *417*, 746–750. https://doi.org/10.1038/nature00802.

6. Dunny, G.M., Brown, B.L., and Clewell, D.B. (1978). Induced cell aggregation and mating in *Streptococcus faecalis*: evidence for a bacterial sex pheromone. Proc Natl Acad Sci U S A *75*, 3479–3483. https://doi.org/10.1073/pnas.75.7.3479.

7. Ladjouzi, R., Bizzini, A., Lebreton, F., Sauvageot, N., Rincé, A., Benachour, A., and Hartke, A. (2013). Analysis of the tolerance of pathogenic enterococci and *Staphylococcus aureus* to cell wall active antibiotics. Journal of Antimicrobial Chemotherapy *68*, 2083–2091. https://doi.org/10.1093/jac/dkt157.

8. Harrington, S.M., Ross, T.L., Gebo, K.A., and Merz, W.G. (2004). Vancomycin Resistance, esp, and Strain Relatedness: a 1-Year Study of Enterococcal Bacteremia. Journal of Clinical Microbiology *42*, 5895–5898. https://doi.org/10.1128/jcm.42.12.5895-5898.2004.

9. Leenhouts, K., Buist, G., Bolhuis, A., ten Berge, A., Kiel, J., Mierau, I., Dabrowska, M., Venema, G., and Kok, J. (1996). A general system for generating unlabelled gene replacements in bacterial chromosomes. Mol Gen Genet *253*, 217–224. https://doi.org/10.1007/s004380050315.

10. Jönsson, M., Saleihan, Z., Nes, I.F., and Holo, H. (2009). Construction and Characterization of Three Lactate Dehydrogenase-Negative *Enterococcus faecalis* V583 Mutants. Appl Environ Microbiol *75*, 4901–4903. https://doi.org/10.1128/AEM.00344-09.

11. Zhang, X., Vrijenhoek, J.E.P., Bonten, M.J.M., Willems, R.J.L., and van Schaik, W. (2011). A genetic element present on megaplasmids allows *Enterococcus faecium* to use raffinose as carbon source. Environ Microbiol *13*, 518–528. https://doi.org/10.1111/j.1462-2920.2010.02355.x.
